# Sigma-1 and Sigma-2 receptor ligands induce apoptosis and autophagy but have opposite effect on cell proliferation in uveal melanoma

**DOI:** 10.18632/oncotarget.19556

**Published:** 2017-07-25

**Authors:** Lucia Longhitano, Carlo Castruccio Castracani, Daniele Tibullo, Roberto Avola, Maria Viola, Giuliano Russo, Orazio Prezzavento, Agostino Marrazzo, Emanuele Amata, Michele Reibaldi, Antonio Longo, Andrea Russo, Nunziatina Laura Parrinello, Giovanni Li Volti

**Affiliations:** ^1^ Department of Biomedical and Biotechnological Sciences, University of Catania, Catania, Italy; ^2^ Department of Ophthalmology, University of Catania, Catania, Italy; ^3^ Regional Reference Center for Rare Diseases, Clinical Division of Hematology and Transplantation, PO Ferrarotto Hospital, Azienda Ospedaliera-Universitaria Policlinico-Vittorio Emanuele, Via Citelli, Catania, Italy; ^4^ Department of Drug Sciences, University of Catania, Catania, Italy; ^5^ Euromediterranean Institute of Science and Technology, Palermo, Italy

**Keywords:** uveal melanoma, sigma receptors, apoptosis, autophagy, (+)-pentazocine

## Abstract

Uveal melanoma is the most common primary intraocular tumor in adults, with about 1200–1500 new cases occurring per year in the United States. Metastasis is a frequent occurrence in uveal melanoma, and outcomes are poor once distant spread occurs and no clinically significant chemotherapeutic protocol is so far available. The aim of the present study was to test the effect of various σ_1_ and σ_2_ receptor ligands as a possible pharmacological strategy for this rare tumor. Human uveal melanoma cells (92.1) were treated with various concentrations of different σ_2_ ligands (haloperidol and haloperidol metabolite II) and σ_1_ ligand ((+)-pentazocine) at various concentrations (1, 10 and 25 μM) and time points (0, 4 h, 8 h, 24 h and 48 h). Cell proliferation and migration were evaluated respectively by continuous cell monitoring by xCELLigence analysis, clonogenic assay and wound healing. Apoptosis and autophagy were also measured by cytofluorimetric and microscopy analysis. Our results showed that σ_2_ receptor ligands significantly reduced cell proliferation whereas (+)-pentazocine exhibited opposite results. All tested ligands showed significant decrease in cell migration. Interestingly, both σ_1_ and σ_2_ receptor ligands showed significant increase of autophagy and apoptosis at all concentrations. Taken all together these results suggest that sigma receptors mediates opposite biological effects but they also share common pharmacological effect on apoptosis and autophagy in uveal melanoma. In conclusion, these data provide the first evidence that sigma receptors may represent a “druggable” target to develop new chemotherapic agent for uveal melanoma.

## INTRODUCTION

**Figure 1:**
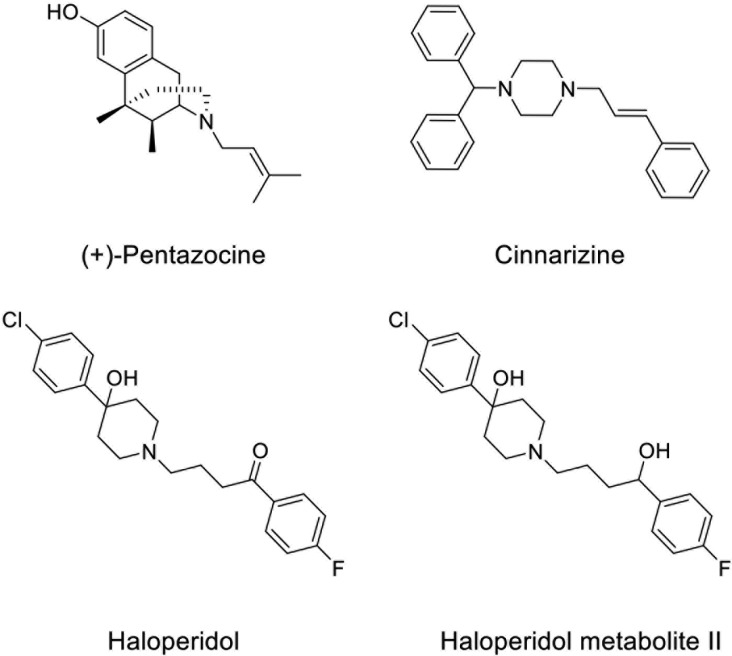
Chemical structures of σ receptor ligands (+)-Pentazocine, Cinnarizine, Haloperidol, and Haloperidol metabolite II

Uveal melanoma is the most common primary intraocular tumor in adults, with about 1200–1500 new cases occurring per year in the United States [1, 2]. Although both uveal and cutaneous melanomas arise from melanocytes, uveal melanoma is biologically and genetically distinct from the more common cutaneous melanoma. Metastasis is a frequent occurrence in uveal melanoma, and outcomes are poor once distant spread occurs. It is estimated that 40–50% of uveal melanoma patients will die of metastatic disease, even with early diagnosis, proper treatment, and close follow-up [3]. By far the most common site of metastasis is the liver, reported in 87% of metastasis cases [4]. The management of localized uveal melanoma can be divided into globe-preserving therapy or enucleation. Globe-preserving therapies can broadly be classified into radiation, surgical, and laser therapy. The majority of primary uveal melanoma lesions in the United States are treated with plaque brachytherapy based upon results of the Collaborative Ocular Melanoma Study (COMS) trial, which randomized patients with medium-sized choroidal melanomas to primary therapy with ^125^I brachytherapy versus enucleation. No difference was observed in mortality between the two groups up to 15 years of follow-up [5]. Furthermore, no chemotherapeutic regimen or immunotherapy was demonstrated to be effective and at this stage they are not considered a clinically significant alternative to radiation or surgery. Bioinformatics as well as other methods are regression, classification or statistical methods used in the chemical and biological sciences helping in predict variables or in understanding patterns [6–8]. To this regard, a recent report using the L1000CDS^2^ web-based utility was able to predict small molecules and drugs targeting uveal melanoma gene signature [9]. In this bioinformatics study, cinnarizine (Figure 1), an anti-histaminic drug used for motion sickness, was proposed as a promising drug for the treatment of metastatic uveal melanoma [9]. Since cinnarizine significantly inhibited (+)-[^3^H]- (+)-pentazocine binding (IC_50_ = 162 ± 28 nM), and a QSAR study predicted a high Sigma-2 (σ_2_) receptor affinity, we hypothesized that Sigma (σ) receptors could play also a role in uveal melanoma progression [10–13].

Sigma receptors, first introduced as subtypes of the opioid receptors, are now considered unique class of proteins. There exist two different σ receptors namely Sigma-1 (σ_1_) and σ_2_ receptors distinguished by structure, biological functions and ligands sensitivity. Indeed, σ_1_ binding sites display high affinity for dextro benzomorphan enantiomers like (+)-pentazocine, while opioid receptors bind levo isomers [14–16]. Small molecules that bind to the σ receptor (σ ligands or σ-modulating drugs) have many potential functions and are used in diagnostic tumor imaging [17–23]. Such compounds are also able to modify the growth rates of human cancer cell lines both *in vitro* and *in vivo*. Several lines of evidence suggest that both receptors are involved in different biological functions including cell proliferation and survival. Interestingly, previous reports showed that σ_1_ receptor is involved in apoptosis because of its location at the mitochondria-associated membranes [24–31]. Similarly, a previous study showed that both sigma receptors are also associated to autophagy in multiple myeloma cells [32].

Taken all together, these observations suggest that σ receptor biological significance is not fully elucidated and may be dependent on the experimental conditions. Therefore, the aim of the present study was to evaluate the pharmacological effects of σ receptor ligands with different pharmacological profile such as haloperidol, haloperidol metabolite II, and (+)-pentazocine (Figure 1, Table 1) on apoptosis and autophagy in uveal melanoma. Our results showed that all tested compounds promote apoptosis and autophagy whereas the σ_1_ receptor agonist (+)-pentazocine has opposite effects on cell proliferation with respect to haloperidol and haloperidol metabolite II, which possess σ_1_ antagonist/σ_2_ ligand profiles.

**Table 1:** Binding profile of haloperidol, haloperidol metabolite II, and (+)-pentazocine

	K*i* (nM) ± S.E.M.			
	σ_1_	σ_2_	D_2_	D_3_
Haloperidol^a^	2.7 ± 0.5	17.0 ± 1.5	2.5 ± 0.7	6.1 ± 1.5
Haloperidol metabolite II^a^	2.9 ± 0.8	2.4 ± 0.5	241 ± 38	1024 ± 217
(+)-Pentazocine^b^	5.0 ± 1.0	1824 ± 36	−	−

^a^Reference [28].

^b^Reference [52].

## RESULTS

### Effect of pharmacological treatments on cell proliferation and migration

**Figure 2:**
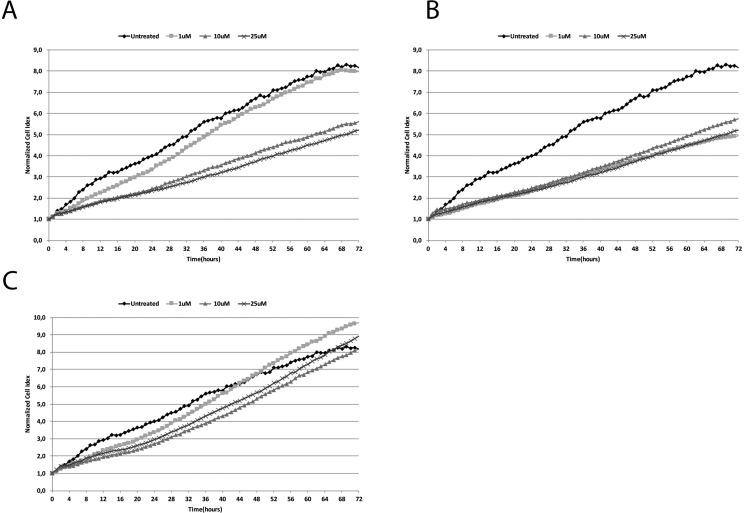
Real time cell proliferation monitoring by xCELLigence system following treatments with (**A**) (+)-Pentazocine, (**B**) Haloperidol and (**C**) Haloperidol metabolite II. Cell index values were normalized at the time of pharmacological treatments in order to obtain a normalized cell index. Each line is expressing the average of four different experiments.

Our results showed that haloperidol treatment resulted in a dose dependent effect on cell proliferation (Figure 2A). Interestingly, haloperidol treatment at 1 μM resulted in a significant increase (*p* < 0.01) of cell proliferation when compared to control over a 72 h period. Interestingly all the other concentrations (10, and 25 μM) resulted in a significant (*p* < 0.01) decrease of cell proliferation when compared to control. Conversely, haloperidol metabolite II (Figure 2B) showed a significant decrease of cell proliferation at all concentrations when compared to control (*p* < 0.01). By contrast, continuous cell proliferation analysis showed that (+)-pentazocine (Figure 2C) resulted in a dose dependent increase of cell proliferation when compared to control. In particular, the concentrations exhibiting higher proliferative effects were 1 and 25 μM (*p* < 0.01). These two concentrations exhibited their effect following 24 h treatment whereas 10 μM was able to induce proliferation at a lower extent following 48 h.

**Figure 3:**
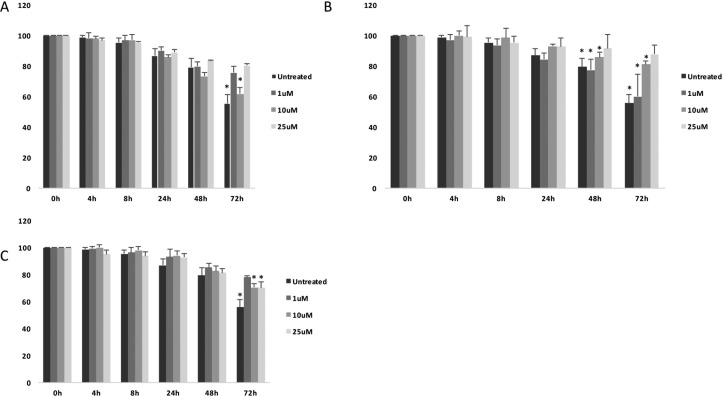
Cell migration analysis following treatments with (**A**) (+)-Pentazocine, (**B**) Haloperidol and (**C**) Haloperidol metabolite II. Values are presented as percentage of the open wound following 4, 8, 24, 48 and 72 hours (wound at time 0 was assumed as 100% and used as control). Values are expressed as the mean ± SD of four different experiments. (*p < 0.01 vs control).

Interestingly, all tested compounds showed significant decrease of cell migration as measured by wound healing assay (Figure 3A–3C).

### Effect of pharmacological treatments on colony formation capacity

**Figure 4:**
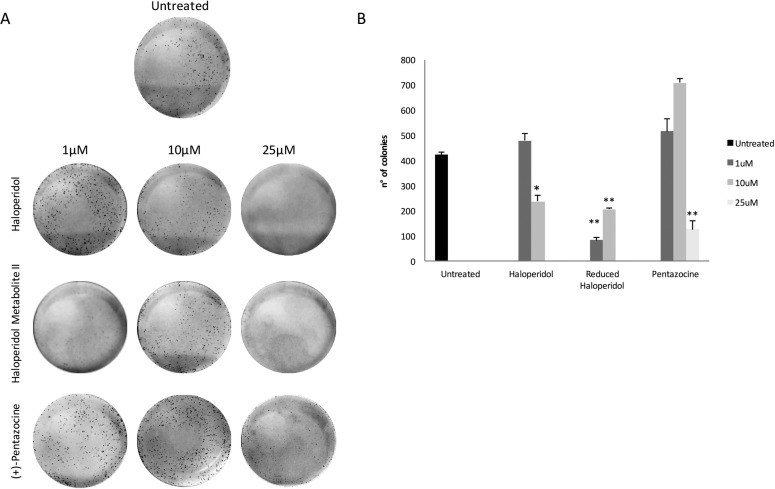
Colony formation capacity following treatments with (+)-Pentazocine, Haloperidol and Haloperidol metabolite II (**A**) Images are representative of four separate experiments and (**B**) number of colonies were manually counted and presented as the mean ± SD of four independent experiments. (*p < 0.01 vs control).

These results were further confirmed in part by clonogenic assay (Figure 4A and 4B) showing that haloperidol was able to induce colonies formation at 1 μM whereas had an opposite effect at 10 and 25 μM (*p* < 0.05). Similarly, all haloperidol metabolite II concentrations were able to decrease colonies formations when compared to control (Figure 4A and 4B). By contrast, (+)-pentazocine treatment resulted in a significant increase in colonies formation at 1 and 10 μM whereas it exhibited opposite results at 25 μM (Figure 4A and 4B).

### Effect of pharmacological treatments on cell autophagy

**Figure 5:**
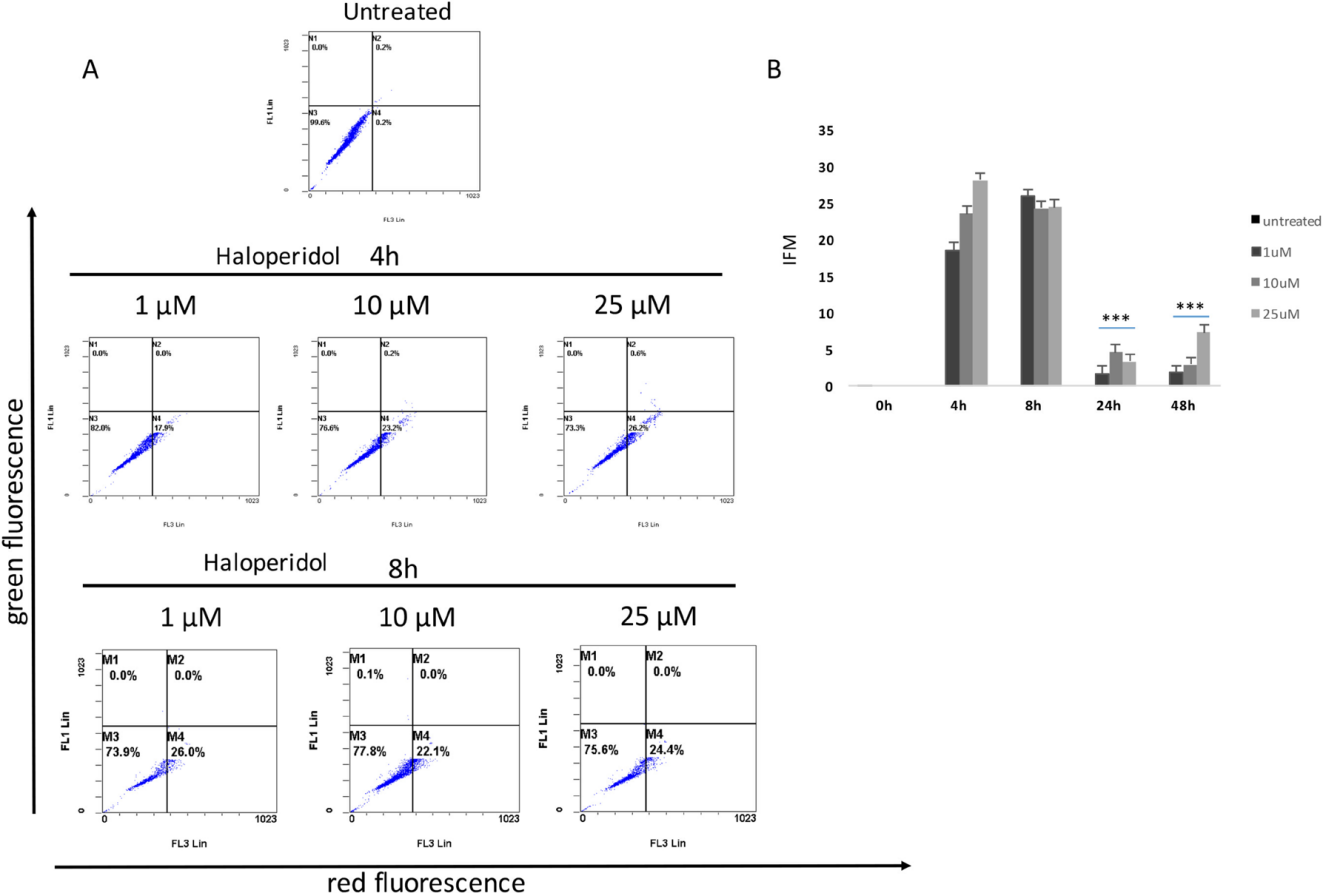
Cytofluorimetric analysis of cell autophagy following Haloperidol treatment at different concentrations and time points (**A**) Blots are representative of three independent experiments; (**B**) percentage of autophagic cells are presented as the mean ± SD of three independent experiments. (*p < 0.01 vs control).

**Figure 6:**
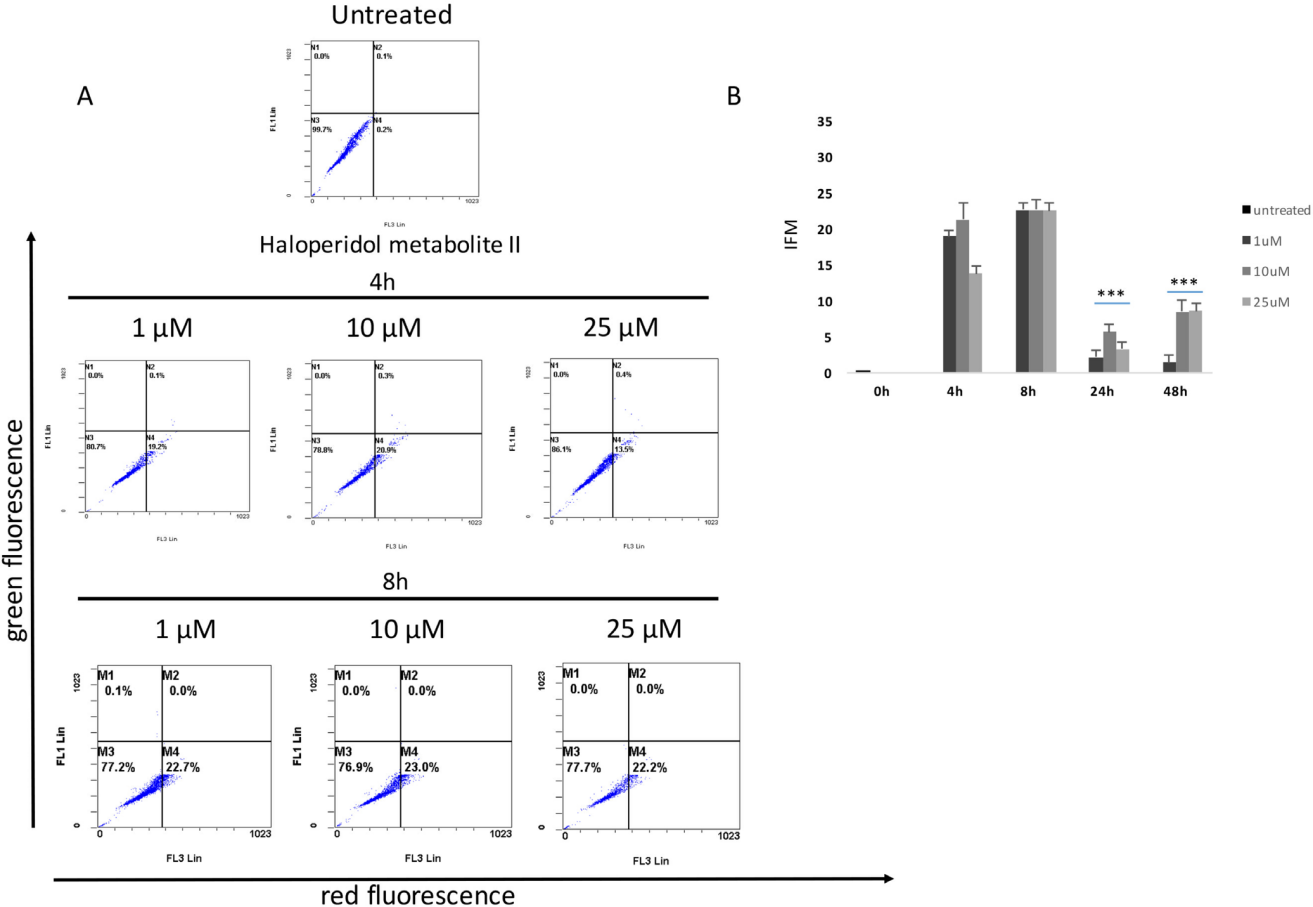
Cytofluorimetric analysis of cell autophagy following Haloperidol metabolite II treatment at different concentrations and time points (**A**) Blots are representative of three independent experiments; (**B**) percentage of autophagic cells are presented as the mean ± SD of three independent experiments. (*p < 0.01 vs control).

**Figure 7:**
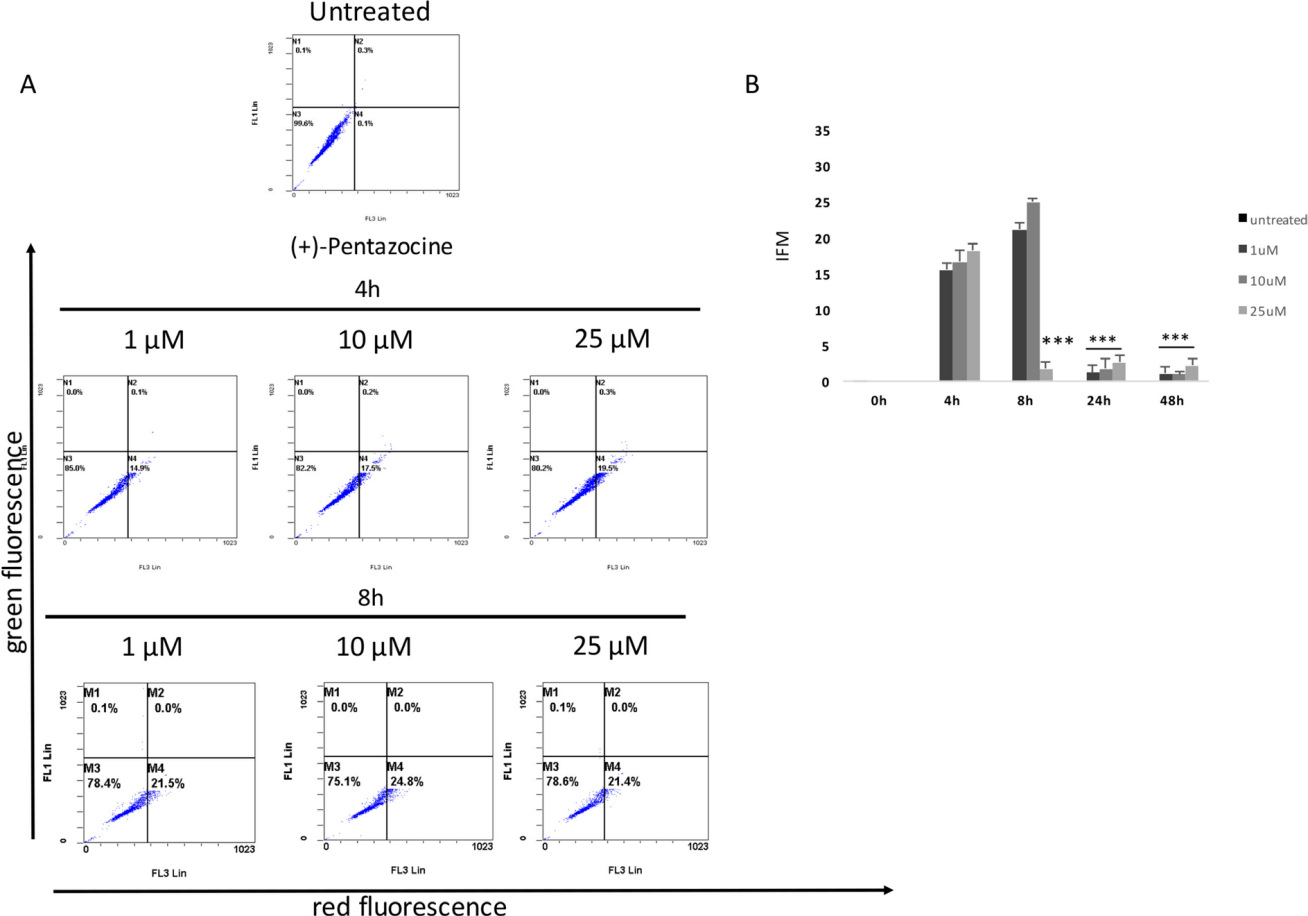
Cytofluorimetric analysis of cell autophagy following (+)-pentazocine treatment at different concentrations and time points (**A**) Blots are representative of three independent experiments; (**B**) percentage of autophagic cells are presented as the mean ± SD of three independent experiments. (*p < 0.01 vs control).

**Figure 8:**
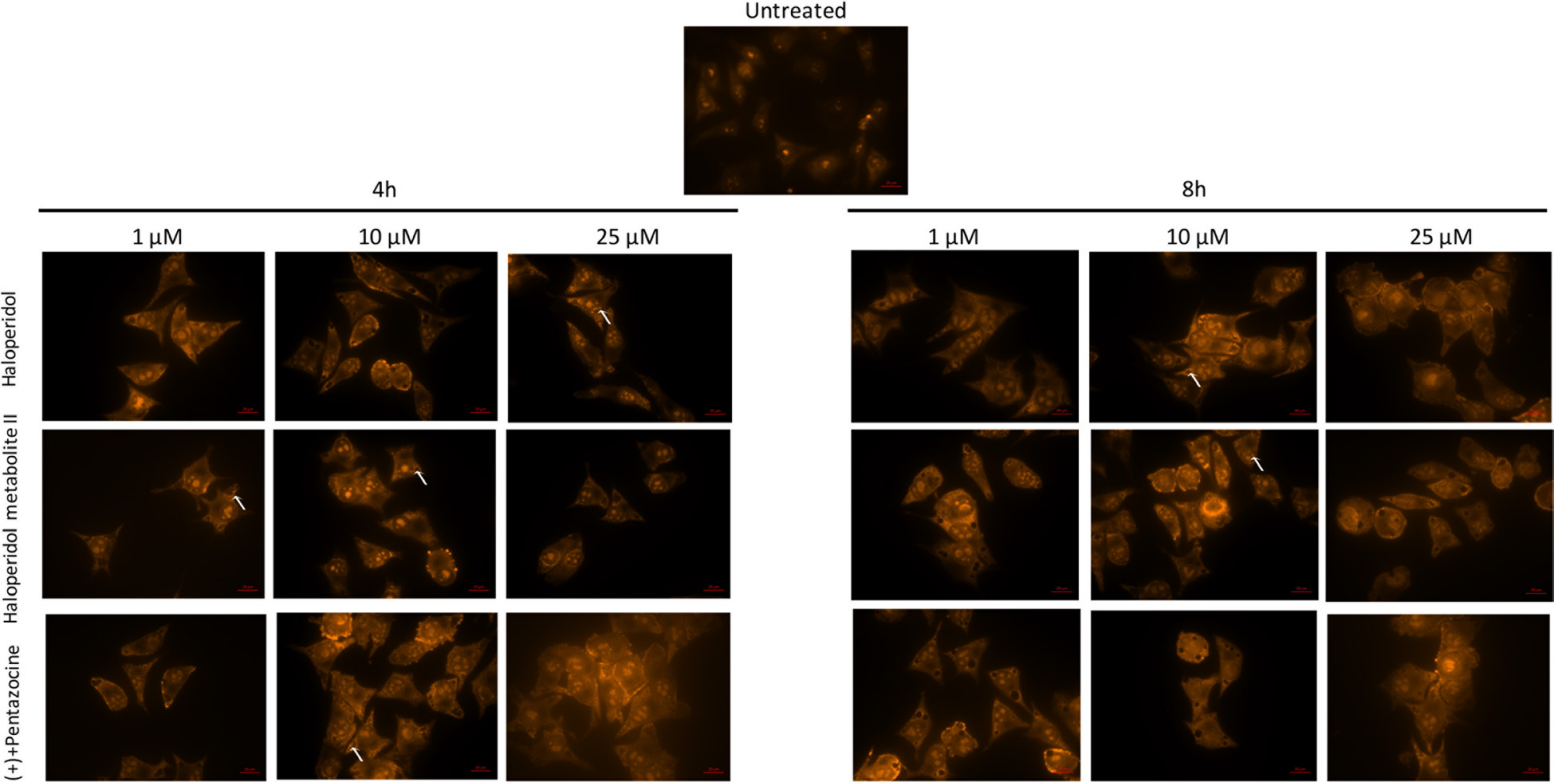
Microscopy analysis of cell autophagy following various pharmacological treatments and time points The protonated form of acridine orange accumulates in acidic compartments and forms aggregates, which are characterized by red/orange fluorescence. Arrows indicate autophagic vacuoles. Images were obtained with a 40× magnification.

Our results showed that all pharmacological treatments significantly increased cell autophagy. In particular, haloperidol treatment resulted in a significant dose and time dependent increase of autophagy peaking at 4 h and 8 h (Figure 5A, 5B and Figure 8). Haloperidol metabolite II exhibited also a pro-autophagic activity peaking at 4 h and 8 h, however we did not observe a dose and time dependent effects of various tested concentrations (Figure 6A, 6B and Figure 8). Finally, (+)-pentazocine treatment resulted in a significant dose and time dependent increase of autophagy peaking at 4 h and 8 h (Figure 7A, 7B and Figure 8).

### Effect of pharmacological treatments on cell apoptosis

**Figure 9:**
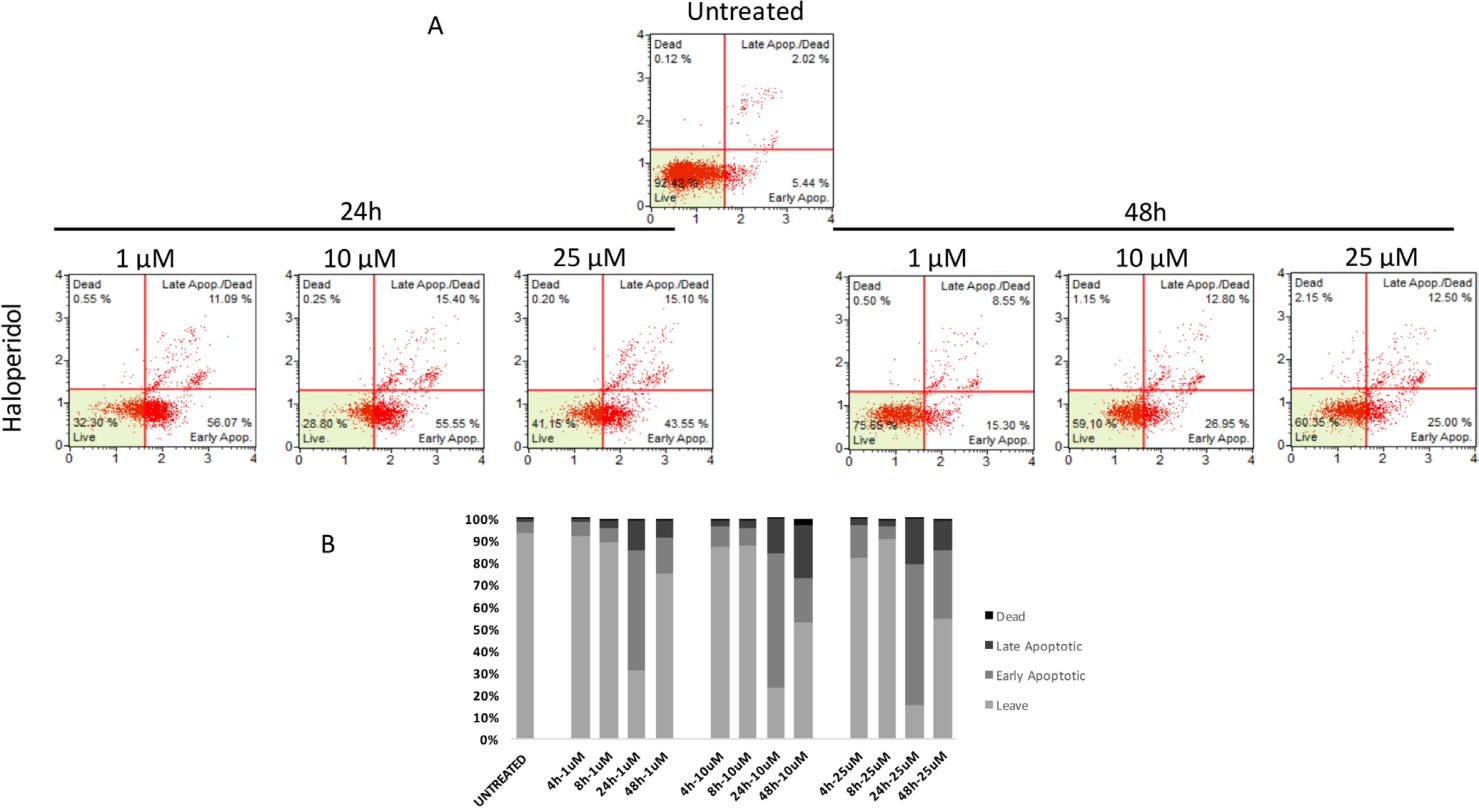
Cytofluorimetric analysis of cell apoptosis following Haloperidol treatment at different concentrations and time points (**A**) Blots are representative of three independent experiments; (**B**) percentage of viable, early and late apoptotic cells and necrotic cells are presented as the mean ± SD of three independent experiments.

**Figure 10:**
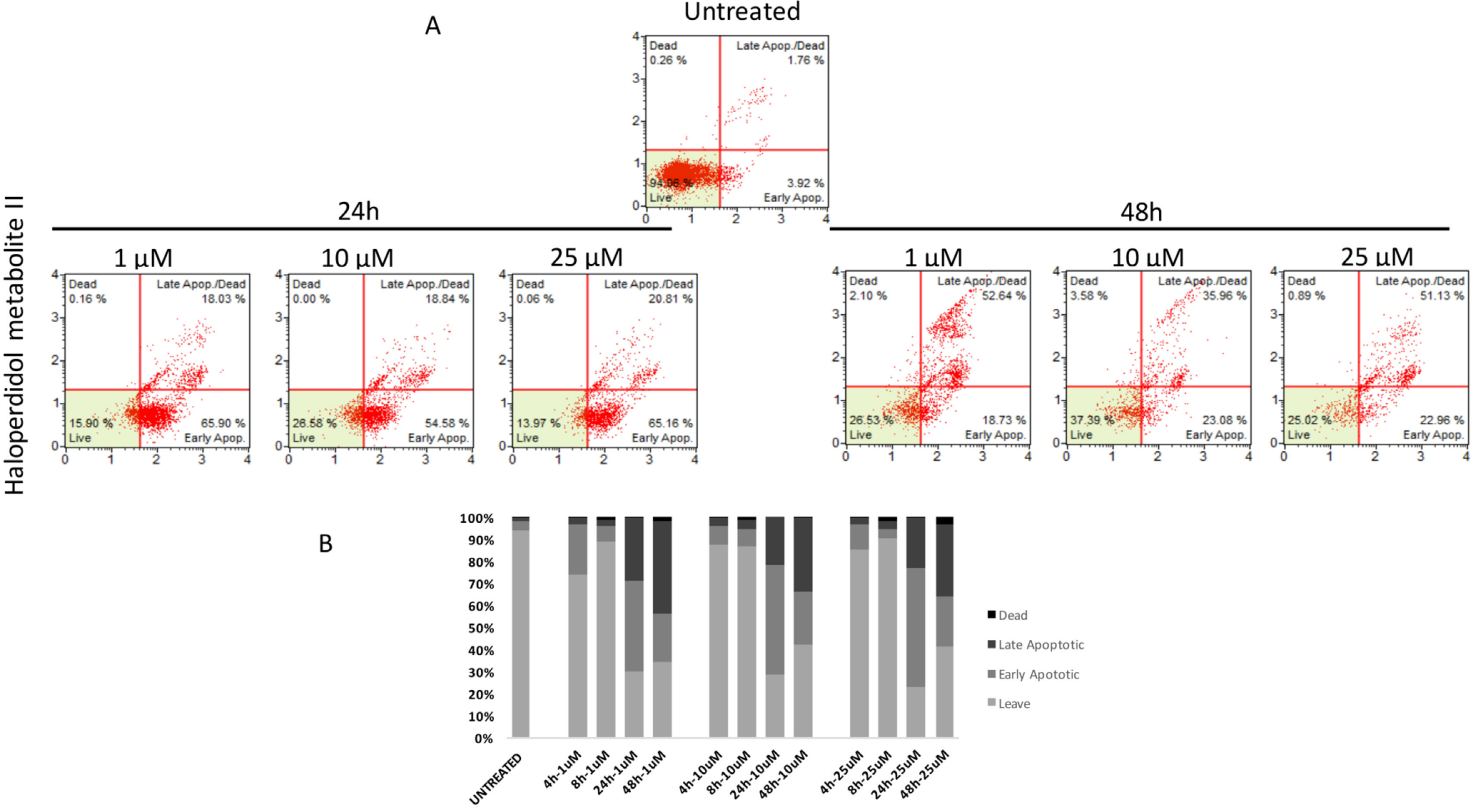
Cytofluorimetric analysis of cell apoptosis following Haloperidol metabolite II treatment at different concentrations and time points (**A**) Blots are representative of three independent experiments; (**B**) percentage of viable, early and late apoptotic cells and necrotic cells are presented as the mean ± SD of three independent experiments.

**Figure 11:**
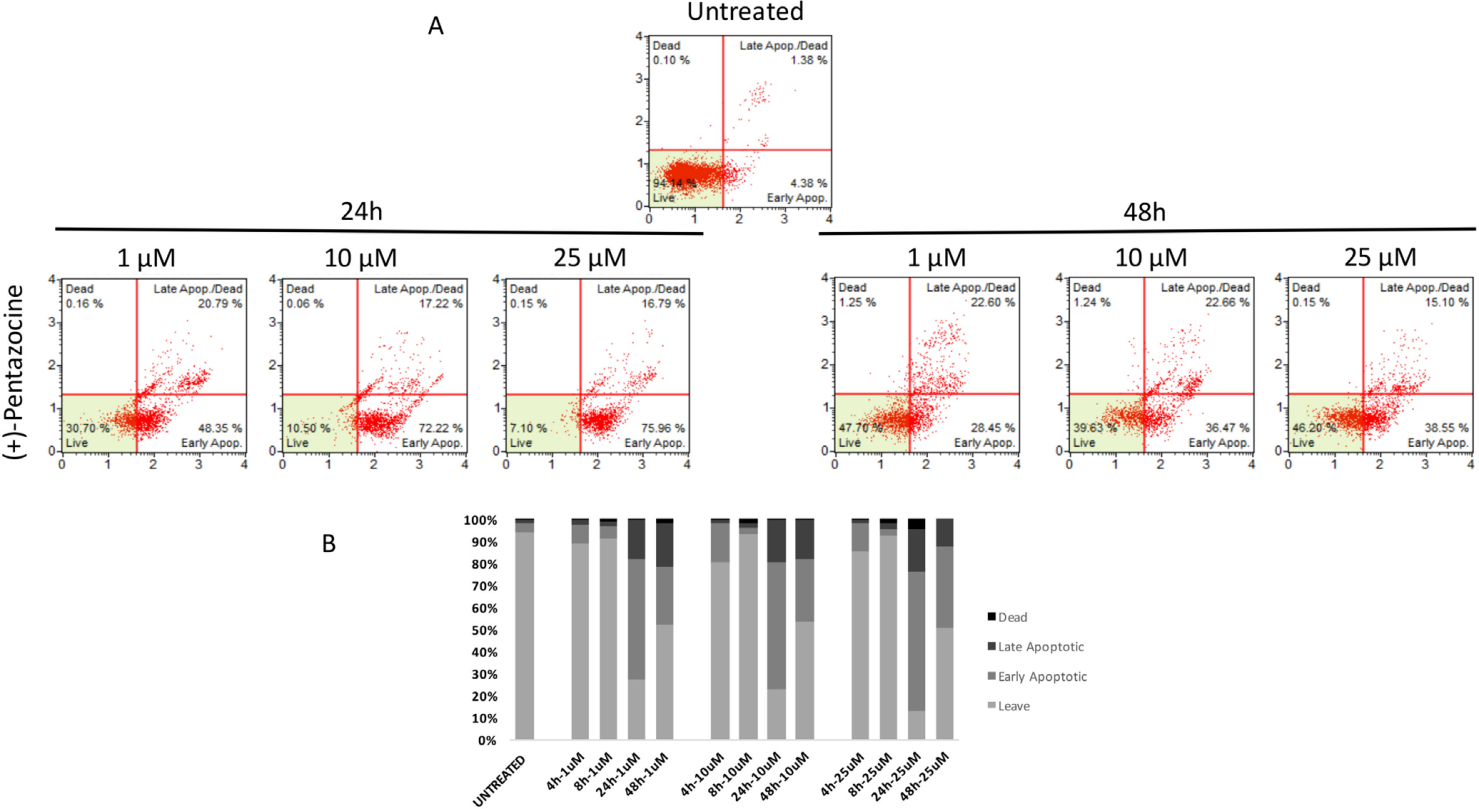
Cytofluorimetric analysis of cell apoptosis following (+)-Pentazocine treatment at different concentrations and time points (**A**) Blots are representative of three independent experiments; (**B**) percentage of viable, early and late apoptotic cells and necrotic cells are presented as the mean ± SD of three independent experiments.

Similarly to autophagy, all pharmacological treatments induced apoptosis at all tested concentrations even though such effect was evident at later time points when compared to autophagy (24 h, and 48 h). In particular, haloperidol was apoptotic at all tested concentrations peaking at 24 h (Figure 9A and 9B). In addition, haloperidol metabolite II induced apoptosis at all tested concentrations peaking at 24 h and exhibited increased late apoptotic cell death at 48 h compared to control and haloperidol (*p* < 0.01 for both) (Figure 10A and 10B). Finally, (+)-pentazocine exhibited an apoptotic profile peaking at 24 h and where it was significantly higher at 10 and 25 μM (*p* < 0.01 compared to control and 1 μM) (Figure 11A and 11B).

## DISCUSSION

Uveal melanoma is a rare subset of melanoma, representing approximately 3–5% of all melanomas and with an incidence of approximately six per million per year in the United States. Although considered a rare tumor, uveal melanoma is the most common primary ocular malignancy in adults and accounts for 85–95% of all ocular melanoma cases. Despite excellent rates of local disease control with surgery or radiotherapy, nearly 50% of patients with uveal melanoma will develop metastatic disease within 15 years from initial diagnosis. Therefore the aim of the present study was to evaluate the “druggability” of σ receptors as possible target for uveal melanoma treatment.

In the first set of experiment we showed that prototypical σ ligands such as (+)-pentazocine (a putative σ_1_ agonist activity) or haloperidol and its reduced metabolite (with σ_1_ antagonist and σ_2_ agonist properties) exhibited different effect on cell proliferation compared to σ_1_ agonist. In particular, the σ_1_ agonist (+)-pentazocine, induced cancer cell proliferation whereas haloperidol and haloperidol metabolite II inhibited cancer growth. Noteworthy, lowest concentration of haloperidol (1 μM) treatment resulted in a significant increase of cell proliferation compared to the other tested concentrations or its metabolite. To this regard, different receptor affinity should be taken into due account for haloperidol and its metabolite. Indeed, haloperidol exhibits a high affinity for both σ receptor subtypes as well as for dopaminergic, adrenergic, serotoninergic and histaminergic receptors [33] whereas haloperidol metabolite II has significant higher affinity and selectivity for σ receptor. Therefore, it is conceivable that haloperidol at low concentrations may exhibit additional non-specific effects.

Our results are consistent with previous studies showing that σ_2_ receptor ligands are associated with proliferation inhibition of cancer cells [34, 35] whereas under our experimental conditions σ_1_ receptor ligand treatment resulted in an increased cell proliferation. These results were partially confirmed by clonogenic assay, which however showed a significant reduction of colony formation capacity following 25 μM (+)-pentazocine treatment. This discrepancy may be related to the different experimental conditions used for the two assays used. In particular, live continuous cell monitoring with xCELLingence analysis allows cell proliferation to be assessed over 72 h time frame, whereas for clonogenic assay, cell are grown at very low confluency and for longer time (10 days). Therefore, it is not possible to exclude that high concentrations of (+)-pentazocine may have different effects on cell proliferation following chronic treatment. Our results are of particular interest, since previous reports showed similar effect of σ_1_ and σ_2_ ligands on cell proliferation whereas we are showing that in uveal melanoma they may have opposite effects on cell proliferation. However, since this is the first report characterizing the σ receptor system in uveal melanoma, we cannot exclude that this might be a cell specific effect. Consistently with this hypothesis is the evidence that even the same receptor mediates opposite pharmacological effects under various experimental conditions. Futhermore, it should be taken into due account that haloperidol and its metabolite II, beside being σ_2_ ligands, share common σ_1_ antagonist activity. In particular, previous reports suggested that σ_1_ may serve a chaperone molecule, possesses various oligomerization states and different intracellular localization compared to σ_2_ receptor [36, 37]. As far as concern cell migration, our data are consistent with previous reports showing that both sigma receptor ligands significantly reduces cell migration under various experimental conditions [28, 38, 39].

Furthermore, we showed that both σ_1_ ligand and mixed σ_1_ antagonist/σ_2_ agonists share the same effects on autophagy and apoptosis. Several chemotherapeutic agents have been shown to induce autophagy [40]. However, in many cases it remains unclear whether cell death occurs by autophagy, whether cell death is associated with autophagy, or whether autophagy is a survival response to cytotoxic chemotherapy [41–44]. Emerging data suggest that autophagy participates in integrated responses to cellular stress that determine cell death versus survival. Interestingly, we showed that all tested σ receptor ligands induce autophagy following 4 h and 8 h treatments as measured by cytofluorimetric and microscopy analysis. Our results are consistent with previous reports showing that both σ receptors mediate autophagy under various experimental conditions. In particular, under our experimental conditions, autophagy may represent a mechanism related to cell toxicity rather than a resistance mechanism since apoptosis occurs at later time points (24 h, and 48 h). Our data are consistent with a previous study showing that both sigma receptors are also associated to autophagy in multiple myeloma cells [32]. Consistently with a previous report [45], our data demonstrate that σ receptor ligands induce cell death by multiple signaling pathways and that both autophagy and apoptosis coexist, even though at different time points, following σ receptor ligands treatment. Finally, both cell death mechanisms seem to be cell specific since under different experimental conditions σ receptor ligands do not induce either apoptosis or autophagy [46].

Taken all together, our data suggest that both σ receptors are able to induce apoptosis and autophagy in uveal melanoma but they exert different effects on cell proliferation. In conclusion, σ receptors represent a valuable target for uveal melanoma treatment and additional studies are now warranted in order to fully elucidate the molecular mechanisms downstream receptor activation in order to elucidate their possible translation into a clinical setting.

## MATERIALS AND METHODS

### Cell culture and pharmacological treatments

Human uveal melanoma cells (92.1) were purchased from ATCC Company (Milan, Italy). Cells were suspended in RPMI1640 culture medium containing 10% fetal bovine serum (FBS), 100 U/mL penicillin, and 100 U/mL streptomycin). At 80% confluency, cells were passaged using trypsin-EDTA solution (0.05% trypsin and 0.02% EDTA) [47]. (+)-Pentazocine (Sigma–Aldrich, Milan, Italy), haloperidol, and haloperidol metabolite II were added separately to cell culture of all experiments at different final concentrations of 1, 10, and 25 μM.

### Clonogenic assay

Colony assays performed by seeding cells in 6 wells plates at low density (3000 cells/well) and allowing growth for 10 days. Colonies were fixed, stained with crystal violet and counted.

### Annexin V and dead cell evaluation by cytofluorimetric analysis

Cell apoptosis was evaluated by Muse™ Annexin V & Dead cell kit (Catalog No. MCH100105, Millipore, Milan, Italy) according to the manufacture’s guidelines. Briefly, 100 μl of the Muse™ Annexin V & Dead Cell Reagent to 100 μl of cell suspension. Such preparation was mixed thoroughly by vortexing at a medium speed for 3–5 s and samples were allowed to stain for 20 min at room temperature in the dark. Samples were read by Muse™ Cell Analyzer (Millipore).

### Effects of pharmacological treatments on cell migration

Cell migration was studied by employing the “wound healing” assay. Briefly, cells were seeded in 24 wells dishes and cultured until confluence. Cells were treated with vehicle, (+)-pentazocine, haloperidol or haloperidol metabolite II and were then scraped with a 200 μl micropipette tip and monitored at 0 h, 4 h, 8 h, 24, 48 h, and 72 h. The uncovered wound area was measured and quantified at different intervals with ImageJ 1.37v (NIH).

### Real time cell proliferation monitoring by xCELLigence system

xCELLigence experiments were performed using the RTCA (Real-Time Cell Analyzer) DP (Dual Plate) instrument according to manufacturers’ instructions (Roche Applied Science, Mannheim, Germany and ACEA Biosciences, San Diego, CA). The RTCA DP Instrument includes three main components: (i) RTCA DP Analyzer, which is placed inside a humidified incubator maintained at 37°C and 5% CO_2_, (ii) RTCA Control Unit with RTCA Software preinstalled, and (iii) E-Plate 16 for proliferation assay. First, the optimal seeding number was determined by cell titration and growth experiments. After seeding the optimal cell number (5000 cells/well), cells were automatically monitored every 15 min [48, 49]. Optimal cell number was determined in a preliminary set of experiments (data not shown) in order to obtain a significant cell index value and a constant cell growth during the entire duration of the experiment. Cells were treated with the compounds about 8h after seeding, when the cells were in the log growth phase.

### Cytofluorimetric and microscopy analysis of autophagy

Human uveal melanoma cell line was incubated with 3 μL of acridine orange (Sigma, St. Louis, MO, USA) at a final concentration of 1 μM for 15 min at 37°C at room temperature in the dark. Following washing with phosphate buffer solution (PBS), the acidic vacuoles were detected using a fluorescence microscope. Autophagic cells contained bright red cytoplasmic particles. Formation of acidic vesicular organelles was quantified also by flow cytometry following acridine orange staining. Briefly, cells were fixed with methanol for 3 minutes and rinsed with ware before acridine orange (1 μM in acetic acid) staining for 15 minutes. Finally, cell were rinsed with water andcells were analyzed by cytometry. In particular, the appropriate isotopic control was also included and labeled cells were acquired using a Beckman Coulter FC-500 flow cytometer [50, 51].

### Statistical analysis

The data were expressed as the means ± SD. Statistical analysis was performed via one-way analysis of variance (ANOVA) using SPSS11.0 software. *p* < 0.05 was considered to be significant.

## Abbreviations

(COMS): Collaborative Ocular Melanoma Study
(FBS): Fetal Bovine Serum
(NSCLC): Non-small cell lung carcinoma
(PBS): Phosphate Buffer Solution
(RTCA): Real-Time Cell Analyzer
