# Correction: Sigma-1 and Sigma-2 receptor ligands induce apoptosis and autophagy but have opposite effect on cell proliferation in uveal melanoma

**DOI:** 10.18632/oncotarget.28519

**Published:** 2024-03-14

**Authors:** Lucia Longhitano, Carlo Castruccio Castracani, Daniele Tibullo, Roberto Avola, Maria Viola, Giuliano Russo, Orazio Prezzavento, Agostino Marrazzo, Emanuele Amata, Michele Reibaldi, Antonio Longo, Andrea Russo, Nunziatina Laura Parrinello, Giovanni Li Volti

**Affiliations:** ^1^Department of Biomedical and Biotechnological Sciences, University of Catania, Catania, Italy; ^2^Department of Ophthalmology, University of Catania, Catania, Italy; ^3^Regional Reference Center for Rare Diseases, Clinical Division of Hematology and Transplantation, PO Ferrarotto Hospital, Azienda Ospedaliera-Universitaria Policlinico-Vittorio Emanuele, Via Citelli, Catania, Italy; ^4^Department of Drug Sciences, University of Catania, Catania, Italy; ^5^Euromediterranean Institute of Science and Technology, Palermo, Italy

**This article has been corrected:** In Figure 4A, the 1st plate image in the 10 μM column is an accidental duplicate of the 2nd plate image in that same column. The corrected Figure 4, obtained using the original data, is shown below. The authors declare that these corrections do not change the results or conclusions of this paper.

Original article: Oncotarget. 2017; 8:91099–91111. 91099-91111. https://doi.org/10.18632/oncotarget.19556

**Figure 4:**
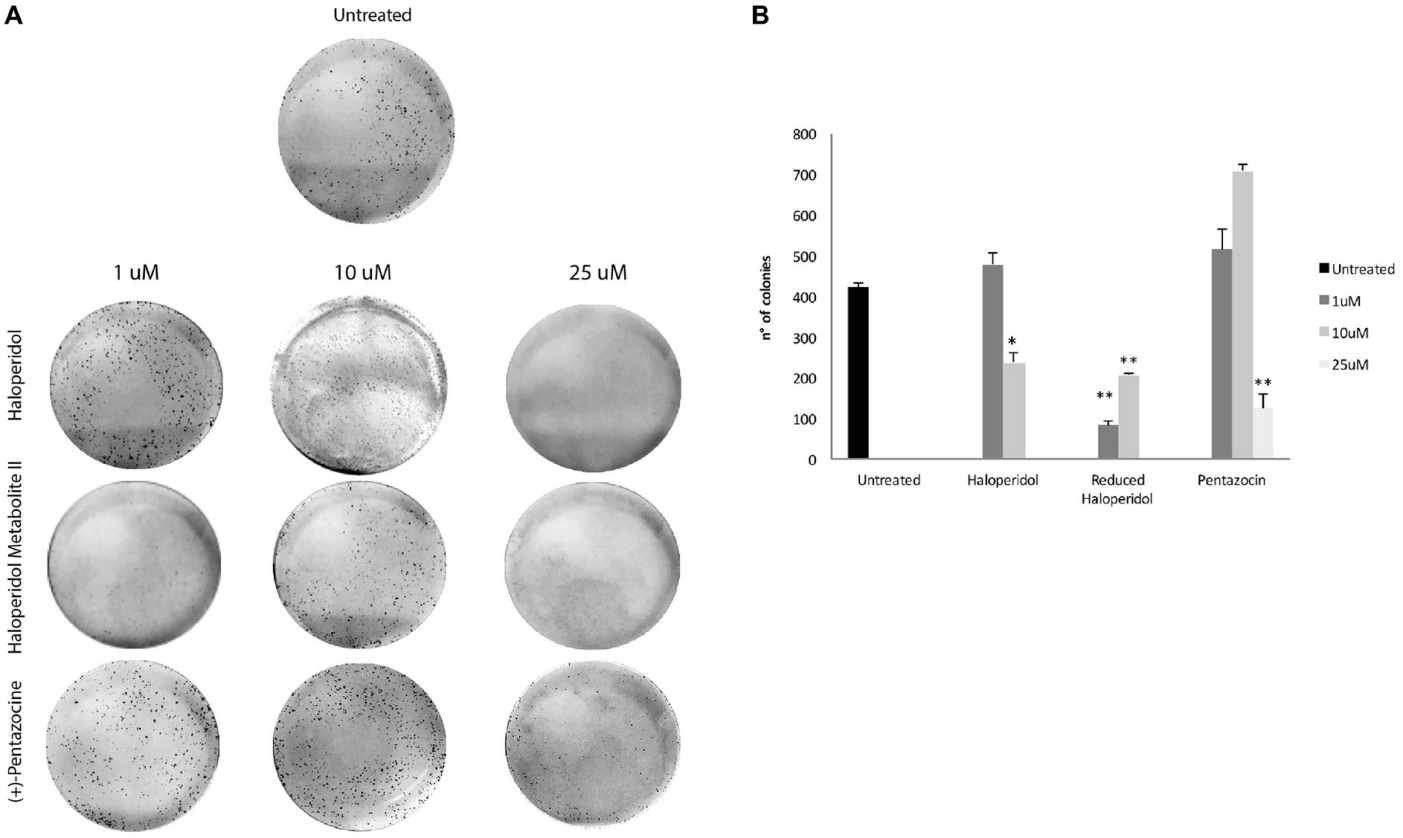
Colony formation capacity following treatments with (+)-Pentazocine, Haloperidol and Haloperidol metabolite II. (**A**) Images are representative of four separate experiments and (**B**) number of colonies were manually counted and presented as the mean ± SD of four independent experiments. (^*^ *p* < 0.01 vs. control).

